# American Association for Study and Prevention of Infant Mortality

**Published:** 1910-09

**Authors:** 


### AMERICAN ASSOCIATION FOR STUDY AND PREVENTION OF INFANT MORTALITY.

A special report on birth registration is being prepared under the direction of Dr. Casey L. Wilbur, Chief of the Division of Vital Statistics of the Bureau of the Census, for the first annual meeting of the American Association for Study and Pervention of Infant Mortality which will be held in Baltimore in November. The report of the committee on birth registration will be presented at the session on municipal, State and Federal prevention of which Dr. Wm. H. Welch is chairman. The members of the committee on birth registration include in addition to Dr. Wilbur:

Dr. Wilber R. Batt, Commissioner of Vital Statistics, Harrisburg, Pa.

Dr. Charles V. Chapin, Commissioner of Health, Providence, Rhode Island.

Dr. John S. Fulton, Secretary-General Int. Cong. on Hygiene and Demography, Washington.D. C.

Dr. John N. Hurty, Secretary State Board of Health, Indianapolis, Ind.

Dr. Wm. C. Woodward, Health Officer, Washington, D. C.

The meeting will open with a general session on November

9th. On the 10th and 11th there will be four special sessions as follows:

*Municipal, State and Federal Prevention.*

Chairman—Dr. Wm. H. Welch, Johns Hopkins Medical School, Baltimore, Md.

Secretary—Dr. John S. Fulton, Sec.-General Int. Cong. on Hygiene and Demography, Washington, D. C.

*Medical Prevention.*

Chairman—Dr. L. Emmett Holt, 14 W. 55th street, New York City.

Secretary—Dr. Philip Van Ingen, 125 East 71st street, New York City.

*Educational Prevention.*

Chairman—Dr. Helen C. Putnam, chairman of the committee to investigate the teaching of hygiene, appointed by the American Academy of Medicine, 1903. Providence, R. I.

Secretary—Prof. Abby L. Marlatt, Department of Home Economics, University of Wisconsin, Madison, Wisconsin.

*Philanthropic Prevention.*

Chairman—Dr. Hastings H. Hart, Director Department of Child-Helping, Russell Sage Foundation, 105 East 23d street, New York City.

Secretary—Mr. Sherman C. Kingsley, Superintendent United Charities, Chicago, Ill.

The officers of the association are:

President—Dr. J. H. Mason Knox, Jr., Baltimore, Md.

President-elect—Prof. Chas. Richmond Henderson, Chicago.

Vice-President—Prof. C. E. A. Winslow, Boston.

Vice-President—Mr. Homer Folks, New York City.

Secretary—Dr. Linnaeus E. La Fetra, Editor, *Archives of Pediatrics,* New York City.

Treasurer—Mr. Austin McLanahan, care Alex. Brown & Sons, Baltimore.

Every section of the country is represented in the directorate. The headquarters of the association are in the Medical and Chirurgical Faculty Building, 1211 Cathedral street, Baltimore, Maryland.

For information or circular write to the executive secretary, Gertrude B. Knipp.

